# Identification and analysis of the genetic integrity of different types of rice resources through SSR markers

**DOI:** 10.1038/s41598-023-29514-y

**Published:** 2023-02-10

**Authors:** Huiying Zhou, Shuhui Li, Jin Liu, Jiaxiao Hu, Si Le, Maomao Li

**Affiliations:** Jiangxi Academy of Agricultural Sciences, Nanchang, 330200 People’s Republic of China

**Keywords:** Molecular biology, Plant sciences

## Abstract

Seed aging is the key factor leading to the loss of genetic integrity. In this study, the seeds of Dongxiang wild rice, Xianggu, 9194 and Nipponbare were kept in a plant incubator with constant temperature and humidity for artificial aging treatment. The genetic integrity of germplasm resources with different germination gradients were analyzed using 44 SSR markers. The results suggested that different accessions could be ranked in order of aging resistance from highest to lowest as common wild rice > Xianggu > 9194 > Nipponbare. In order to maintain the genetic diversity of rice, the population size for reproduction and regeneration should be between 60 and 140. After aging, the number of polymorphic alleles, the number of specific single plant, the ratio of polymorphic bands, the number of alleles, the number of effective alleles, gene diversity index and Shannon index of different accessions all decreased with the decrease of germination rate. The germination rate of 60% was the critical value to maintain genetic integrity. Besides, the genetic integrity of eighteen SSR markers was rapidly lost or significantly increased. The regions of these markers were closely related to seed viability or genetic integrity. This study provides a theoretical basis for determining the population size for reproduction and regeneration and the critical value of germination rate of rice resources.

## Introduction

Genetic integrity refers to the original genetic composition of germplasm resources. The protection of genetic integrity refers to the maintenance of the complete genetic structure of germplasm populations in the process of reproduction, regeneration or long-term conservation^1,2^. Changes in the genetic integrity of germplasm refer to the gradual loss of genetic integrity with the decrease of seed germination rate and viability during storage, including the differential differentiation between the progeny populations and the parent populations of germplasm resources after reproduction and regeneration^3^. The genetic integrity of germplasm is affected by many factors, such as seed aging, population size for reproduction and regeneration, and pollination methods^4^. To maintain the genetic integrity of germplasm is to maximize genetic similarity in the process of reproduction and regeneration, and to minimize genetic variation during the long-term conservation^5^. The aging of germplasm resources in storage will decrease seed germination rate, cause natural selection and genetic drift of the genetic genotype, and finally result in the loss of genetic integrity^6^. In short, it is of great scientific to study the effects of seed aging on the genetic integrity of germplasm resources.

As seed longevity is short in the natural conditions, low-temperature conservation and room-temperature drying are mainly used to prolong seed longevity^7^. Although these two methods can effectively extend seed longevity, they cannot prevent decline in seed viability or death of the seeds^8^. China has established more than forty medium- and long-term gene banks of different types and preserved more than 1 million accessions of germplasm resources^9^. Seeds stored in gene banks will gradually lose their germination ability, which is called natural aging. When the germination rate of stored seeds decreases to a certain lower limit, the germplasm resources must be reproduced and regenerated^10^. The critical value of germination rate standard for the reproduction and regeneration of crop germplasm resources is from 60 to 85% in China, but from 50 to 75% in countries such as the United States, Britain, and India^11,12^. Li et al.^13^ analyzed the purity of 2794 soybean cultivars or lines by using fifty-nine SSR markers. They suggest that the population size should be kept larger during the propagation for those lower-purity accessions compared with high-purity accessions; otherwise the low frequent genotypes are easy to be lost. Sun et al.^14^ studied the effect of regenerate population on the genetic integrity of sesame germplasm and showed that the genetic integrity could be maintained when the population size of released cultivar reached 35 to 40. Due to the obvious differences in seed structure and inclusions among different types of crops, the difficulty of aging as well as reproduction and regeneration varies significantly. Therefore, it is of great significance to determine the optimal population size and critical value of germination rate for reproduction to maintain the genetic integrity of germplasm resources.

Maintaining the genetic integrity of stored crop germplasm resources is the core issue for the safe conservation and regeneration of germplasm resources. In particular, the identification of the purity or authenticity of varieties at the molecular level is critical to the maintenance of the genetic integrity of germplasm^15^. Methods for identifying the genetic integrity of germplasm resources mainly include morphological markers, cellular markers, biochemical markers and molecular markers. Among them, morphological markers and biochemical markers have obvious limitations as they are easily affected by environment, time-consuming and complicated to operate^16^. With the development of molecular biotechnology, molecular marker technologies such as RFLP, AFLP and SSR provide efficient and simple methods for exploring the genetic integrity of germplasm resources at the molecular level. Molecular markers can directly detect differences and changes in DNA without being restricted by environmental conditions^17^. In recent years, molecular marker detection technology has become a powerful supplement to morphological, cellular and biochemical markers in the identification of genetic integrity and is widely used in testing the genetic integrity of wheat, corn, cotton, rye and other crops^18^^,^^19^. Since there are few studies on the identification of the genetic integrity of different rice accessions, the method of using molecular technology for detecting genetic integrity is still unclear. In this study, common wild rice, rice landraces, indica cultivars and japonica cultivars were used as experimental materials. Germplasm populations with different germination rates were obtained through artificial aging. SSR markers were used to identify the genetic integrity of rice resources with different germination rates. And the critical value of germination rate and population size for reproduction and regeneration were determined in order to maintain the genetic integrity of rice resources. Thus, this study aims to provide a theoretical basis for the maintenance of genetic integrity and safe conservation of germplasm resources.

## Results

### Change rules of seed viability

The aim of the aging test was to determine the aging resistance of different accessions and to obtain the germplasm populations with different germination rates. The actual germination parameters of all samples are presented in Table 1. The initial germination rates of the aged seeds were all above 90%. There were significant differences in the treatment time of the samples with different germination rates such as 80–85%, 60–70%, 50%, 30% and 0. Significant differences were observed in the aging resistance of different accessions. The aging resistance of Dongxiang wild rice was the best as the seed viability was completely lost in twenty-four days after aging test. In contrast, Nipponbare had the lowest level of aging resistance as the seed viability was completely lost in fourteen days.

**Table 1:** Germination rates of different accessions after aging treatment.

Germination rates (%)	Aging treatment days (d)			
	Dongxiang wild rice	Xianggu	9194	Nipponbare
≥ 90	0	0	0	0
80–85	9	9	8	6
60–70	12	12	10	8
50	15	14	12	10
≤ 30	17	16	14	12
0	24	18	16	14

### Seed phenotypic characters

**Figure 1:**
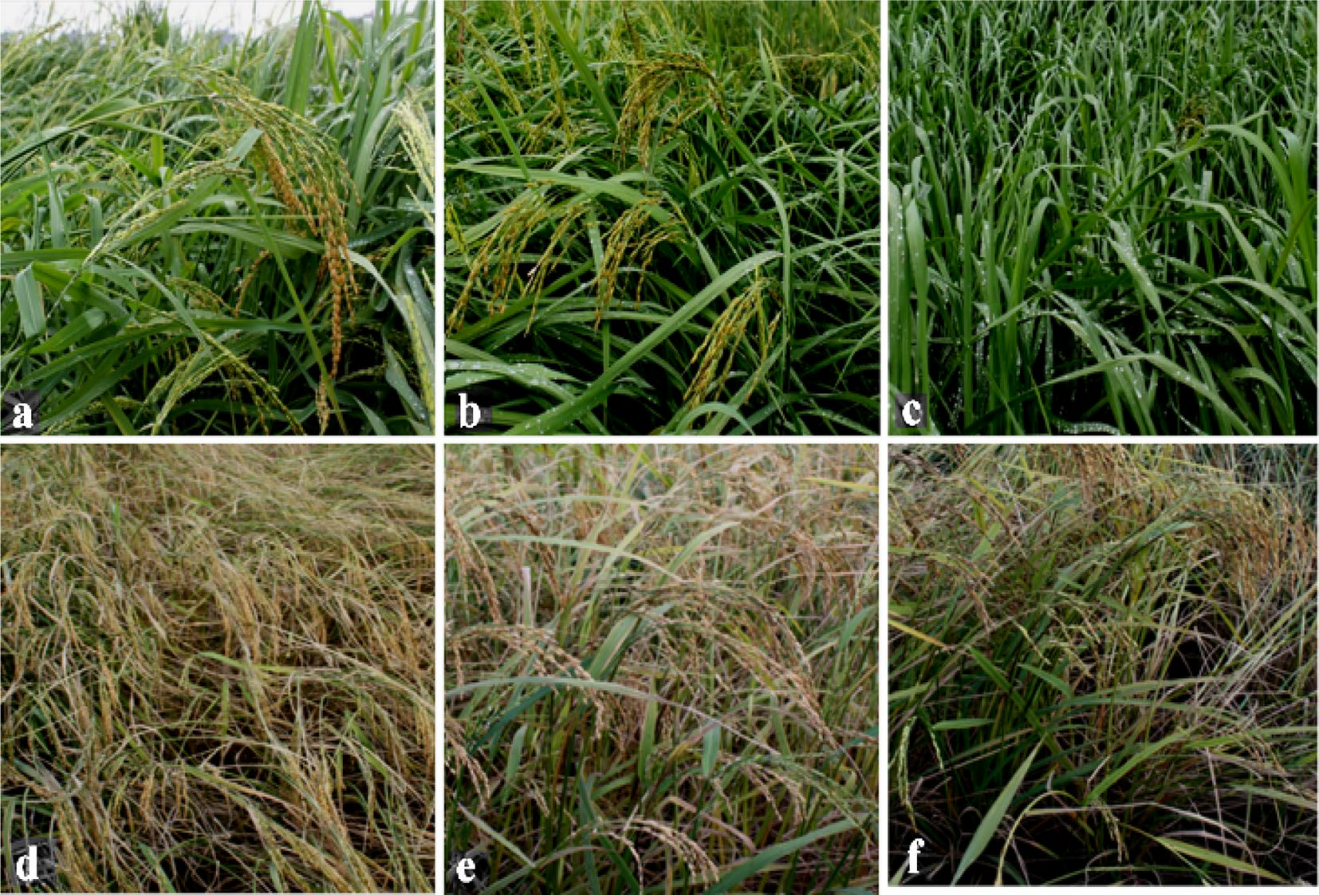
The phenotypic characters of rice landraces under different germination rates. (**a**–**c**) represents the phenotype characters at heading stage with the germination rates of 80–85%, 65% and 30%, respectively. (**d**–**f**) represents the phenotype characters at filling stage with the germination rates of 80–85%, 65% and 30%, respectively.

Xianggu is a typical landrace with rich genetic diversity. The phenotypes of most of the individual plants in Xianggu were medium maturity, hemp husks and short awn, while that of some individual plants were late maturity, hemp husks and long awn or early maturity, yellow husks and no awn (Fig. 1). Significant differences were observed in the germination rate, the number of effective panicle, heading stage, initial heading stage, full heading stage and mature stage of the same variety with different germination rates (Table 2). The main phenotypic characters of different accessions did not change obviously except Xianggu. Significant differences were not found between the phenotypic characters of test materials with germination rates of 90% and 80–85%.

**Table 2:** Germination rate and germinating time of different varieties under different germination gradients.

Germination rates (%)	Varieties			
	Dongxiang wild rice	Xianggu	9194	Nipponbare
≥ 90	4d germination rate 97%	3d germination rate 98%	3d germination rate 98%	3d germination rate 97%
80–85	4d germination rate 97%	3d germination rate 98%	3d germination rate 98%	3d germination rate 97%
60–70	6d germination rate 24%	4d germination rate 63%	4d germination rate 60%	4d germination rate 60%
50	8d germination rate 46%	6d germination rate 47%	6d germination rate 45%	6d germination rate 46%
≤ 30	13d germination rate 20%	6d germination rate 24%	6d germination rate 24%	6d germination rate 28%

The days in the table is calculated from the germination time.

### Population size for reproduction and regeneration

In order to determine the change of phenotypic characters as the germination rate decreases after aging test, Xianggu, with obvious differences in phenotypic traits, was selected to study the population size for reproduction and regeneration. The germination rate and seed viability declined as the aging level increased. The population size for reproduction had a significant effect on the frequency of individual plants with late maturity, hemp husks and long awn or early maturity, yellow husks and no awn (Table 3). When the germination rate was 80–85%, the minimum population size for reproduction was 40 plants if one plant exhibits those phenotypes, 80 plants when two plants exhibit those phenotypes and 100 plants if three plants exhibit those phenotypes. When the germination rate was 60–70% and 50%, the minimum population size for reproduction was 60 plants when one plant exhibits those phenotypes, 80 plants if two plants exhibit those phenotypes and 160 plants if three plants exhibit those phenotypes. When the germination rate was 30%, the minimum population size for reproduction was 60 plants if one plant exhibits those phenotypes, 140 plants if two plants exhibit that phenotypes and 200 plants when three plants exhibit those phenotypes. Thus, in order to maintain the genetic diversity of rice resources, the population size for reproduction and regeneration should be between 60 and 140. The lower the germination rate, the larger the population size for reproduction and regeneration, otherwise, the population size for reproduction and regeneration could be appropriately reduced.

**Table 3:** Population size for reproduction of Xianggu on the genetic diversity of phenotypic traits with different germination rates.

Population size	Germination rates (%)	Late maturity hemp husk long awns (plant)	Early maturity yellow husks no awns (plant)	Population size	Germination rates (%)	Late maturity hemp husk long awns (plant)	Early maturity yellow husks no awns (plant)
20 plants	≥ 90	0	0	120 plants	≥ 90	4	4
	80–85	0	0		80–85	4	3
	60–70	1	0		60–70	3	2
	50	0	1		50	2	3
	≤ 30	0	0		≤ 30	1	4
40 plants	≥ 90	1	1	140 plants	≥ 90	5	4
	80–85	1	1		80–85	5	3
	60–70	1	0		60–70	3	2
	50	0	1		50	2	3
	≤ 30	0	0		≤ 30	2	4
60 plants	≥ 90	3	1	160 plants	≥ 90	5	4
	80–85	3	1		80–85	5	3
	60–70	3	1		60–70	3	5
	50	2	1		50	3	3
	≤ 30	1	1		≤ 30	2	4
80 plants	≥ 90	3	2	180 plants	≥ 90	5	5
	80–85	3	2		80–85	6	3
	60–70	3	2		60–70	3	5
	50	2	2		50	3	4
	≤ 30	1	3		≤ 30	2	6
100 plants	≥ 90	3	3	200 plants	≥ 90	6	5
	80–85	3	3		80–85	6	4
	60–70	3	2		60–70	3	8
	50	2	2		50	4	6
	≤ 30	1	4		≤ 30	5	7

### The polymorphic primers selected

A total of 650 pairs of SSR primers were selected, which distribute evenly over the whole rice genome. These primers were used to detect the polymorphism of 200 individual plants in the control varieties (Dongxiang wild rice, Xianggu, 9194 and Nipponbare) with the germination rate of 90%. According to PAGE detection, only 44 pairs of primers were polymorphic among the control varieties. Finally, these polymorphic primers were selected for the detection of genetic integrity. The number of the polymorphic primers in Dongxiang wild rice, Xianggu, 9194 and Nipponbare was twenty-five, sixteen, twelve and eight, respectively (Table 4). Among them, eleven pairs of primers, namely RM6, RM24, RM110, RM154, RM175, RM254, RM339, RM351, RM565, RM575 and RM6334, appeared twice in Dongxiang wild rice, Xianggu and 9194. Three pairs of primers, that is RM282, RM296, and RM552 appeared at the same time. The eight pairs of polymorphic primers selected from Nipponbare did not appear in other varieties.

**Table 4:** Polymorphic primers for the genetic integrity in different varieties.

Varieties	SSR markers	Number
Dongxiang wild rice	RM575, RM297, RM318, RM183, RM452, RM327, RM174, RM301, RM262, RM565, RM36, RM282, RM175, RM241, RM153, RM534, RM510, RM162, RM351, RM70, RM339, RM296, RM41, RM552, RM254	25
Xianggu	RM24, RM110, RM406, RM6, RM154, RM565, RM5819, RM282, RM175, RM471, RM351, RM118, RM339, RM296, RM552, RM6334	16
9194	RM575, RM24, RM110, RM6, RM211, RM154, RM282, RM169, RM296, RM552, RM254, RM6334	12
Nipponbare	RM446, RM8, RM1385, RM482, RM545, RM218, RM567, RM6317	8

### The critical value of germination rate

These polymorphic primers were used to detect the populations with germination rates of 80–85%, 60–70%, 50% and 30%, respectively. Significant differences in the genetic integrity of different accessions under different germination rate gradients were observed. The results indicated that the number of polymorphic alleles, the number of specific single plant and the ratio of polymorphic bands decreased with the decrease of germination rate. Indeed, the parameters decreased significantly when the germination rate decreased to 50% or less (Table 5).

**Table 5:** Genetic variation in different varieties after aging treatment.

Varieties	Germination rates (%)	Number of polymorphic alleles	Number of specific single plant	Ratio of polymorphic bands (%)
Dongxiang wild rice	≥ 90	53	32	100.00
	80–85	51	32	96.23
	60–70	49	27	92.45
	50	36	18	67.92
	≤ 30	33	14	62.26
Xianggu	≥ 90	35	20	100.00
	80–85	35	17	100.00
	60–70	33	8	94.28
	50	29	6	82.86
	≤ 30	25	5	71.43
9194	≥ 90	28	20	100.00
	80–85	27	17	96.43
	60–70	25	13	89.29
	50	24	14	85.71
	≤ 30	23	11	82.14
Nipponbare	≥ 90	21	33	100.00
	80–85	21	33	100.00
	60–70	20	20	95.24
	50	17	10	80.95
	≤ 30	16	8	76.19

The number of alleles, the number of effective alleles, gene diversity index and Shannon index of different accessions also decreased with the decrease of seed germination rate. No significant differences were observed except for the Shannon index in Xianggu at a germination rate of 60% or more, and the number of alleles in Nipponbare at a germination rate of 50% or more. When the germination rate decreased to 50% or less, all of the traits showed a significant or extremely significant difference, except the number of effective alleles in Dongxiang wild rice and the number of alleles in 9194. All of the parameters showed an extremely significant difference when the germination rate decreased to 30% (Table 6). The results showed that the genetic integrity of different accessions was better at a germination rate of 80% or above. When the germination rate decreased to 50% or less, the number of alleles, the number of effective alleles, gene diversity index and Shannon index of different accessions decreased rapidly. It was preliminarily suggested that the germination rate of 60–70% was the critical value to maintain genetic integrity.

**Table 6:** Genetic diversity of different varieties with different germination rates.

Varieties	Germination rates (%)	Number of alleles (Na)	Number of effective alleles (Ne)	Gene diversity index (He)	Shannon index (I)
Dongxiang wild rice	≥ 90	2.227 ± 0.408	1.257 ± 0.281	0.991 ± 0.019	0.327 ± 0.193
	80–85	2.124 ± 0.332 ns	1.169 ± 0.258 ns	0.979 ± 0.039 ns	0.231 ± 0.186 ns
	60–70	2.040 ± 0.351 ns	1.051 ± 0.054 ns	0.953 ± 0.039 ns	0.210 ± 0.090 ns
	50	1.446 ± 0.583**	1.023 ± 0.046	0.853 ± 0.045**	0.147 ± 0.075**
	≤ 30	1.320 ± 0.476**	1.010 ± 0.020**	0.821 ± 0.126**	0.093 ± 0.044**
Xianggu	≥ 90	2.188 ± 0.447	1.082 ± 0.111	0.581 ± 0.291	0.148 ± 0.111
	80–85	2.063 ± 0.250 ns	1.074 ± 0.168 ns	0.569 ± 0.075 ns	0.104 ± 0.151 ns
	60–70	2.000 ± 0.500 ns	1.072 ± 0.181 ns	0.557 ± 0.103 ns	0.088 ± 0.164*
	50	1.813 ± 0.544**	1.038 ± 0.109**	0.549 ± 0.099**	0.067 ± 0.133**
	≤ 30	1.500 ± 0.516**	1.023 ± 0.063**	0.532 ± 0.073**	0.041 ± 0.088**
9194	≥ 90	1.929 ± 0.267	1.083 ± 0.056	0.960 ± 0.295	0.154 ± 0.091
	80–85	1.857 ± 0.363 ns	1.067 ± 0.036 ns	0.841 ± 0.049 ns	0.137 ± 0.060 ns
	60–70	1.786 ± 0.426 ns	1.055 ± 0.065 ns	0.791 ± 0.543 ns	0.104 ± 0.102 ns
	50	1.714 ± 0.469 ns	1.043 ± 0. 0320*	0.630 ± 0.308**	0.093 ± 0.064*
	≤ 30	1.643 ± 0.497**	1.032 ± 0.039**	0.616 ± 0.315**	0.070 ± 0.066**
Nipponbare	≥ 90	2.665 ± 0.340	1.188 ± 0.005	0.068 ± 0.013	0.172 ± 0.034
	80–85	2.625 ± 0.744 ns	1.088 ± 0.008 ns	0.063 ± 0.023 ns	0.169 ± 0.024 ns
	60–70	2.500 ± 0.756 ns	1.064 ± 0.050 ns	0.059 ± 0.043 ns	0.139 ± 0.082 ns
	50	2.125 ± 0.641*	1.047 ± 0.042 ns	0.044 ± 0.038 ns	0.105 ± 0.083 ns
	≤ 30	2.00 ± 0.538**	1.026 ± 0.021**	0.025 ± 0.020**	0.065 ± 0.048**

*represents a significant difference at the 5% level, **represents a significant difference at the 1% level, and ns indicates that there is no significant difference. All of the results were compared with the original germination rate.

### Genetic integrity analysis

**Figure 2:**
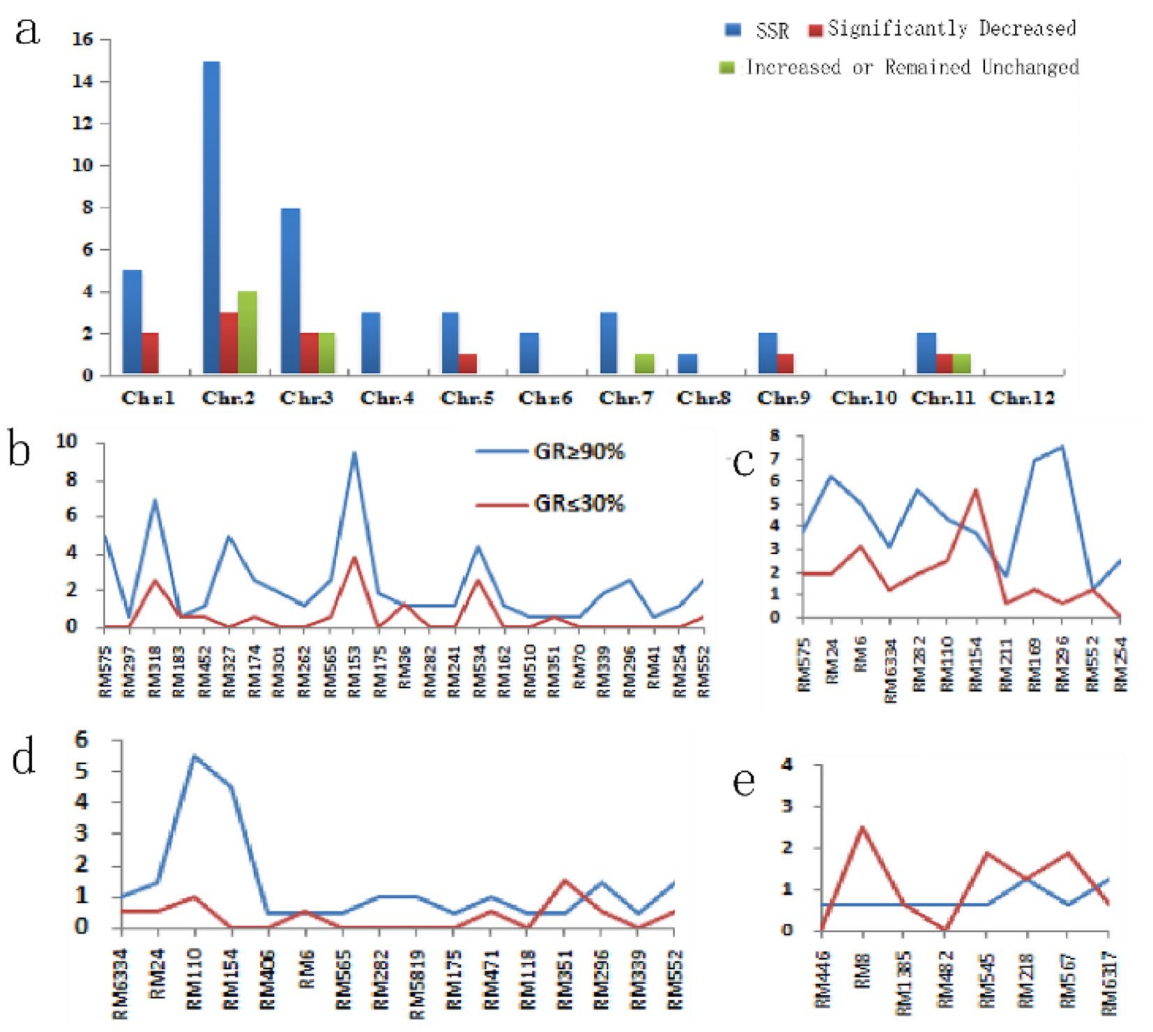
Distribution of SSR markers and variation of polymorphism frequency under different seed viability. (**a**) The distribution of SSR markers and the genetic integrity of different rice types. (**b**, **c**, **d**, **e**) The change of SSR markers in Dongxiang wild rice, Xianggu, 9194 and Nipponbare.

**Figure 3:**
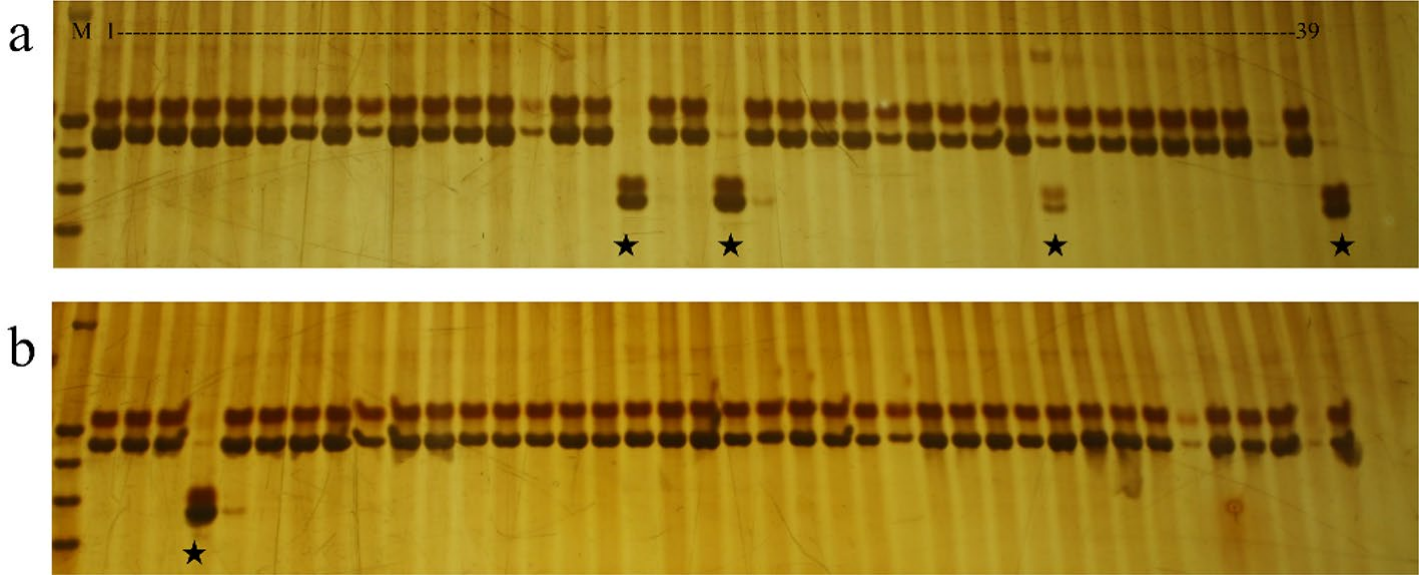
Detection results of RM24 marker in some single plants of Xianggu under different seed viability. (**a**,**b**) represent the seed with the germination rates of 90% and 30%, respectively. ★ represents the specific single plant under SSR marker detection.

In order to determine the characteristics of the selected SSR primers, the chromosome distribution and genetic integrity of the primers were identified and compared. It was found that these primers were unevenly distributed on chromosomes one to nine and eleven (Fig. 2). The number of SSR primers on chromosome two was the highest (fifteen), while the number of SSR primers on chromosomes ten and twelve was zero. Compared with the control, the genetic integrity of SSR primers changed significantly at a germination rate of 30% or less (Fig. 3). The integrity of most SSR primers decreased with the decrease of seed viability. The integrity of ten primers (RM575, RM24, RM110, RM318, RM327, RM282, RM153, RM169, RM296 and RM254) significantly decreased after aging test, while that of eight primers (RM6, RM183, RM8, RM1385, RM36, RM218, RM35 and RM552) significantly increased or remained unchanged. In addition, as three primers (RM282, RM296 and RM552) appeared in Dongxiang wild rice, Xianggu and 9194 at the same time, the chromosomal regions of them may be closely related to genetic integrity. These implied that locations with significant changes in genetic integrity were mainly distributed on chromosomes two and three, which could be related to seed storability or viability.

## Discussion

### Aging resistance characteristics in different accessions

As rice seed is the core and the most critical material in production, it is essential to have a safe and effective conservation system. The aging and deterioration of seeds are inevitable phenomena during storage^20^. Wu et al.^21^ showed that there were significant differences in the aging resistance of rice from different geographical sources. For example, the storability of germplasm from Hunan, Hubei, Sichuan, Anhui, Guangdong, Jiangxi, and Jiangsu was better than that of germplasm from other provinces. Generally, aged seeds can be obtained through natural way and artificial way. Natural aging method can be used to evaluate the storage tolerance of rice by the gradual loss of germination ability under natural storage conditions. Although it is more in line with the natural characteristics of seed storage, it is difficult tobe popularized in practical research because of its long cycle, which takes many months or even years. Since the artificial aging could better simulate the physiological and biochemical changes under natural conditions, researchers often age seeds in an artificial way to accelerate the process and build germination gradient for profound research.

Based on the experimental foundation of artificial treatment of large-scale germplasm resources in the early stage, this study optimized the artificial treatment conditions. In this study, the aging test was run in a plant incubator at a temperature of 43 °C and relative humidity of 85%. Most of the previous studies focused on a single variety while the aging test was run in a plant incubator at a temperature of 40 °C and relative humidity of 100%. Compared with previous studies, this optimized test method with good repeatability was more suitable for aging treatment of different varieties. The aging resistant ability of different accessions under artificial aging was basically consistent with that under natural aging. Through the optimized test method, the germplasm resource groups with different germination rate could be obtained quickly and accurately.

In this study, significant differences were observed in artificially aged seed of different types and different accessions could be ranked in order of their aging resistance from highest to lowest as common wild rice > rice landraces > indica cultivars > japonica cultivars. The aging resistance of Dongxiang wild rice was the best as the seed viability was completely lost in twenty-four days after aging test while that of Nipponbare was the worst as the seed viability was completely lost in fourteen days, which was basically consistent with the previous study.

### Genetic integrity of germplasm resources

The core issue of the safe conservation of germplasm resources is the maintenance of genetic integrityincluding the genotype frequency and alleles during reproduction, regeneration and storage. Fang et al.^22^ showed that the germination rate of oat seeds decreased with the increase of storage time. Compared with natural aging, the genetic parameters of artificially aged population decreased at a higher degree. After aging, the genetic diversity of oat seed decreased, and the genetic integrity was destroyed. Xia et al.^5^ detected the genetic integrity of thirty accessions of soybean germplasms with two or three generations through thirty SSR markers, and the results showed that alleles of twenty-five soybean germplasms did not change while alleles of the other five varieties of soybean germplasms have various degrees of changes. In this study, the number of alleles, the number of specific single plant and the ratio of polymorphic bands decreased with the decrease in seed germination rate. The results showed that the genetic integrity of different accessions was better at a germination rate of 80% or above. When the germination rate decreased to 50% or less, the number of alleles, the number of effective alleles, gene diversity index and Shannon index of different accessions decreased rapidly. It was preliminarily suggested that the germination rate of 60–70% was the critical value to maintain genetic integrity.

In addition, previous studies have shown that a certain number of breeding populations should be guaranteed to maintain the genetic diversity of germplasm resources. There was significantly difference in the population size of different germplasm resources^23,24^. In this study, in order to maintain the genetic diversity of rice resources, the population size for reproduction and regeneration should be between 60 and 140. The lower the germination rate, the larger the population size for reproduction and regeneration, otherwise, the population size for reproduction and regeneration can be appropriately reduced. Therefore, higher germination rate and suitable population size are essential to maintaining the genetic diversity of rice germplasm resources.

As a typical self-crossing crop, rice has a very low rate of outcrossing. There is a high genetic homogeneity of the population, and the genetic structures of individuals are basically the same. Dong et al.^25^ showed that the genetic diversity of the rice landraces in Yunnan was rich. The genetic characteristics of germplasm groups planted for many years would change with environment. Jiang et al.^26^ analyzed the genetic diversity of forty Yuelianggu samples from four villages in Yuanyang Hani's terraced fields by using twenty-four SSR markers and twenty phenotypic traits. The results showed that the same local variety had different genetic diversity when planted in different villages. In this study, SSR markers were used to identify the genetic diversity of different accessions. It showed that there was rich genetic diversity within rice resources, and significant differences were found in the genetic diversity of different types. The genetic diversity of the Dongxiang wild rice population was the highest, while that of the indica cultivars and japonica cultivars were lower. This study also indicated that there were significant differences in the genetic diversity in different accessions. It is necessary to use suitable technology for the reproduction, regeneration and conservation of different accessions.

### Relationship between genetic integrity markers and seed viability

At present, lots of germplasm resources with strong viability and storability have been discovered through research on different types of germplasm resources and genetic groups. Seed viability and storability are complex quantitative traits controlled by multiple genes, and the related markers or quantitative trait locus (QTLs) are located on twelve chromosomes of rice^27,28^. Shigemune et al.^29^ investigated the effects of the QTLs for seed longevity, *qLG-2*, *qLG-4* and *qLG-9*, using chromosome segment substitution lines. Cheng et al.^30^ identified a total of nine additive QTLs and eight epistatic QTLs for seed storability using RILS population. The identified QTLs might be applicable for the improvement of pre-harvest sprouting tolerance by marker-assisted selection in rice. Based on the molecular markers and map-based cloning, genome-wide association study (GWAS), high throughput sequencing, and omics technology, more than twenty-three key genes related to seed vigor have been identified^31^. Dong et al.^32^ identified the QTLs for seed storability using two sets of recombinant inbred lines (RILs). Ten QTLs were detected on chromosomes 1, 2, 3, 4, 6, 8, and 12 in SL-RILs, and a total of 12 QTLs were identified on chromosomes 2, 3, 4, 6, 9, and 10 in SH-RILs.

In this study, a total of forty-four pairs of SSR markers were selected for the detection of genetic integrity. These markers were unevenly distributed on chromosomes one to nine and eleven. Among them, the number of SSR markers on chromosome two was the highest while that of chromosomes ten and twelve were the lowest. The result showed that that there were significant differences in the richness of chromosome genetic integrity of different rice resources. In addition, the integrity of RM154 significantly decreased in Xianggu but significantly increased in 9194, which implied that location with significant changes in genetic integrity could be related to seed storability or viability. Through comparative analysis, this study also found that the genetic integrity of eighteen markers increased or decreased significantly after aging test. Furthermore, three primers RM282, RM296 and RM552 appeared in Dongxiang wild rice, Xianggu and 9194 at the same time, which were located on chromosomes three, nine and eleven, respectively. These implied that these markers were associated with the QTLs related to seed viability and storability, which would be the key locations for maintaining the genetic integrity and seed viability. Thus, in order to provide some excellent genetic resources for the maintenance of genetic integrity of germplasm resources, we still need to use relevant materials to construct genetic populations and explore genes for seed viability and storability.

## Methods

### Experiment materials

In this study, Dongxiang wild rice (*Oryza rufipogon* Griff.), Xianggu (*Oryza sativa* L., landrace, provided by the National Crop Gene Bank in Chinese Academy of Agricultural Sciences), 9194 (*Oryza sativa* L., indica cultivar) and Nipponbare (*Oryza sativa* L., japonica cultivar) were used as experimental materials. In 2015, these materials were planted in the experimental field of Rice Research Institute of Jiangxi Academy of Agricultural Sciences. 4 kg seeds of each accession with high seed viability (that is water content lower than 14% and germination rate higher than 90%) were harvested. In order to break dormancy, the seeds were treated at 45 °C for two days and then stored under constant temperature and low humidity for later use.

### Aging test

Aging test was run with four accessions. 100 g seeds of each variety were maintained in a plant incubator (43 °C and 85% of relative humidity). From the second day, the germination rate of these seeds was measured until it decreased to 0. The aging time for different accessions of the germination rates of 80–85%, 60–70%, 50% and 30% was recorded (Table 1).

### Field experiment

According to the aging time recorded in the prior test, Dongxiang wild rice was aged for 9, 12, 15 and 17 days and the aged seeds with the germination rates of 80–85%, 60–70%, 50% and 30% were obtained. Similarly, Xianggu was aged for 9, 12, 14 and 16 days, 9194 was aged for 8, 10, 12 and 14 days, Nipponbare was aged for 6, 8, 10 and 12 days to obtain their aged seeds with different germination rates. The aged seeds and the control (germination rate ≥ 90%) were kept in the germinator at 25 °C. Then, all of the seedlings were transplanted to the experimental field. The main phenotypic traits such as the number of effective panicles, heading stage, initial heading stage, full heading stage and mature stage of each plant were measured and the fresh leaves were collected to extract DNA.

### Molecular testing

The genomic DNA of rice was extracted by the hexadecyl trimethylammonium bromide (CTAB) method^33^. A total of 650 pairs of SSR primers were selected from the rice gene database (http:// www.gramene.org/). These primers distributed evenly over the whole rice genome and synthesized by Beijing Dingguo Biological Co., Ltd. The PCR reaction was performed after purification. Each 10 μL of the amplification reaction consisted of 1 μL of 10 × PCR buffer, 0.3 μL of the dNTP mixture, 1 μL of primers, 0.1 μL of Taq polymerase, 1 μL of genomic DNA, and sterile molecular biology-grade water for making up the volume. All amplifications were performed under the following conditions: 5 min at 94 °C, followed by 30 s at 94 °C, 30 s at 55 °C and 30 s at 72 °C for 35 cycles and 10 min at 72 °C for a final extension. The PCR products were inspected on 6% polyacrylamide gel electrophoresis (PAGE).

### Analyses of the genetic integrity

The electrophoresis results were compared and revised manually. The present bands were labeled as ‘1’, while absent bands were marked as ‘0’ to establish the data matrix in Excel^34^. Genetic diversity was estimated by the number of alleles (Na), effective number of alleles (Ne), Nei’s genetic diversity index (He) and Shannon’s Information index (I) using POPGENE 32. The significance of differences of Na, Ne, He and I between the same name populations collected in different periods was calculated using SPSS software^4^.

### Statistical analyses

All the data were analyzed using SPSS software to illustrate the influence of ageing on studied parameters. Studied parameters were subjected to one-way ANOVA to test for differences within the species. Values presented are mean ± SD of three replicates. Relationships between various parameters were tested via Pearson’s correlation coefficient test. Significant differences in the mean values were determined at *p* < 0.05^35^.

## Data Availability

All of the material is owned by the authors and/or no permissions are required. The data used to support the findings of this study are available from the corresponding author upon request.
